# Comparing the effect of hydroxyethyl starch 130/0.4 with balanced crystalloid solution on mortality and kidney failure in patients with severe sepsis (6S - Scandinavian Starch for Severe Sepsis/Septic Shock trial): Study protocol, design and rationale for a double-blinded, randomised clinical trial

**DOI:** 10.1186/1745-6215-12-24

**Published:** 2011-01-27

**Authors:** Anders Perner, Nicolai Haase, Jørn Wetterslev, Anders Åneman, Jyrki Tenhunen, Anne Berit Guttormsen, Gudmundur Klemenzson, Frank Pott, Karen Doris Bødker, Per Martin Bådstøløkken, Asger Bendtsen, Peter Søe-Jensen, Hamid Tousi, Morten Bestle, Malgorzata Pawlowicz, Robert Winding, Hans-Henrik Bülow, Claude Kancir, Morten Steensen, Jonas Nielsen, Bjarne Fogh, Kristian R Madsen, Nils H Larsen, Marcela Carlsson, Jørgen Wiis, John Asger Petersen, Susanne Iversen, Ole Schøidt, Siv Leivdal, Pawel Berezowicz, Ville Pettilä, Esko Ruokonen, Pål Klepstad, Sari Karlsson, Maija Kaukonen, Juha Rutanen, Sigurbergur Karason, Anne Lene Kjældgaard, Lars Broksø Holst, Jan Wernerman

**Affiliations:** 1 Department of Intensive Care, Centre of Clinical Intervention Research, Copenhagen University Hospital, Rigshospitalet, Denmark; 2 Copenhagen Trial Unit, Centre of Clinical Intervention Research, Copenhagen University Hospital, Rigshospitalet, Denmark; 3 Department of Intensive Care, Sahlgrenska University Hospital, Göteborg, Sweden; 4 Critical Care Medicine Research Group, Tampere University Hospital, Finland; 5 Department of Intensive Care, Haukeland University Hospital, Bergen, Norway; 6 Department of Intensive Care, Landspitalet, Reykjavik, Iceland; 7 Department of Intensive Care, Bispebjerg Hospital, Copenhagen, Denmark; 8 Department of Intensive Care, Esbjerg Hospital, Denmark; 9 Department of Intensive Care, Gentofte Hospital, Denmark; 10 Department of Intensive Care, Glostrup Hospital, Denmark; 11 Department of Intensive Care, Herlev Hospital, Denmark; 12 Department of Intensive Care, Hillerød Hospital, Denmark; 13 Department of Intensive Care, Hjørring Hospital, Denmark; 14 Department of Intensive Care, Herning Hospital, Denmark; 15 Department of Intensive Care, Holbæk Hospital, Denmark; 16 Department of Intensive Care, Holstebro Hospital, Denmark; 17 Department of Intensive Care, Hvidovre Hospital, Denmark; 18 Department of Intensive Care, Næstved Hospital, Denmark; 19 Department of Intensive Care, Odense University Hospital, Denmark; 20 Department of Intensive Care, Slagelse Hospital, Denmark; 21 Department of Intensive Care, Sønderborg Hospital, Denmark; 22 Department of Intensive Care, Vejle Hospital, Denmark; 23 Department of Intensive Care, Helsinki University Hospital, Finland; 24 Department of Intensive Care, Kuopio University Hospital, Finland; 25 St. Olav's University Hospital, Trondheim, Norway; 26 Department of Intensive Care, Karolinska University Hospital, Sweden

## Abstract

**Background:**

By tradition colloid solutions have been used to obtain fast circulatory stabilisation in shock, but high molecular weight hydroxyethyl starch (HES) may cause acute kidney failure in patients with severe sepsis. Now lower molecular weight HES 130/0.4 is the preferred colloid in Scandinavian intensive care units (ICUs) and 1^st ^choice fluid for patients with severe sepsis. However, HES 130/0.4 is largely unstudied in patients with severe sepsis.

**Methods/Design:**

The 6S trial will randomise 800 patients with severe sepsis in 30 Scandinavian ICUs to masked fluid resuscitation using either 6% HES 130/0.4 in Ringer's acetate or Ringer's acetate alone. The composite endpoint of 90-day mortality or end-stage kidney failure is the primary outcome measure. The secondary outcome measures are severe bleeding or allergic reactions, organ failure, acute kidney failure, days alive without renal replacement therapy or ventilator support and 28-day and 1/2- and one-year mortality. The sample size will allow the detection of a 10% absolute difference between the two groups in the composite endpoint with a power of 80%.

**Discussion:**

The 6S trial will provide important safety and efficacy data on the use of HES 130/0.4 in patients with severe sepsis. The effects on mortality, dialysis-dependency, time on ventilator, bleeding and markers of resuscitation, metabolism, kidney failure, and coagulation will be assessed.

**Trial Registration:**

ClinicalTrials.gov: NCT00962156

## Background

Intravascular fluids are the mainstay treatment for resuscitation of patients with severe sepsis. By tradition, colloids have been used to obtain fast circulatory stabilisation, but there are only few trials with patient-centred outcome measures on fluid resuscitation of septic patients. The Surviving Sepsis Campaign recommends either colloids or crystalloids [1], but high molecular weight hydroxyethyl starch (HES) may cause acute kidney failure (AKF) in patients with severe sepsis as observed in a recent meta-analysis [2]. The three largest trials in this analysis studied HES 200/0.6 (MW in kDa/substitution ratio - hydroxyethyl groups per glucose), but found divergent results with respect to kidney failure with this formulation of starch [3-5]. All these trials had methodological weaknesses [6,7], and two large cohort studies in ICU patients also showed divergent results with respect to the risk of adverse renal effects of starch treatment [8,9].

If HES contributes to AKF in severe sepsis, this is of importance as AKF is an independent risk factor for death in these patients [10-13]. Furthermore, if AKF progresses to end-stage kidney disease, prolonged renal replacement therapy will inflict burden to patients and society.

High molecular weight HES also causes coagulopathy and bleeding and increases the rate of transfusion during major surgery [14], but effects in ICU patients are largely unstudied.

Two Cochrane meta-analyses have been published on colloid use in critically ill patients in general. One compared colloids with crystalloids [15], but there were few trials on HES. Therefore, reliable conclusions cannot be drawn. The other analysis included a comparison between albumin and high molecular weight HES. In this, a relative risk reduction (RRR) greater than 20% could be rejected, but the 14% RRR observed in this analysis with the use of HES could not be rejected [16]. As the effects of albumin and crystalloids are likely to be equal [15], an alternative hypothesis may be that high molecular weight HES reduces the risk of death by 10 - 20% compared to crystalloids.

However the high molecular weight HES is hardly ever used in Scandinavian ICUs, where HES 130/0.4 is the preferred colloid [17] and 1^st ^choice fluid for patients with severe sepsis (preliminary data from the SAFE TRIP study, S Finfer, personal communication) and septic shock [18].

At present there are very limited data on the effects of HES 130/0.4 in septic patients. A single trial has been published, in which 20 patients were randomised to fluid resuscitation with either HES 130/0.4 or albumin [19]. On the other hand, the effects of HES 130/0.4 on coagulation and bleeding may be less pronounced than those observed with HES 200/0.6 at least in the perioperative setting [14].

Taken together, two hypotheses can be put forward. Resuscitation with high molecular weight HES may cause AKF in patients with severe sepsis, or may improve survival by up to 20% when compared to crystalloids. In any case, the low molecular weight HES 130/0.4, which is in wide clinical use, is largely unstudied in septic patients. Therefore, there is an urgent need for trials on HES 130/0.4 in patients with severe sepsis.

### Aims

To assess the effects of HES 130/0.4 compared with a balanced crystalloid solution on mortality and end-stage kidney failure on day 90 in patients with severe sepsis.

## Methods/Design

Multicentre, randomised, double-blinded trial with concealed allocation of septic patients 1:1 to fluid resuscitation using 6% HES 130/0.4 in Ringer's acetate (Tetraspan, B Braun Medical AG, Melsungen, Germany) or Ringer's acetate (Sterofundin, B Braun Medical) stratified by the presence of shock or not [8], haematological malignancy or not [8] and inclusion in a university hospital or not.

### Tetraspan

Tetraspan contains hydroxyethylated starch 60 mg 130/0.42 per ml and the isotonic electrolyte solution of sodium chloride, potassium chloride, calcium chloride, magnesium chloride, sodium acetate and malic acid. It is marketed for the indication 'treatment of imminent or manifest hypovolaemia and shock' in all the Nordic countries (Summary of Product Characteristics for Tetraspan 6%, B Braun Medical).

### Sterofundin

Sterofundin contains the isotonic electrolyte solution of sodium chloride, potassium chloride, calcium chloride, magnesium chloride, sodium acetate and malic acid. It is marketed for the indication 'treatment of extracellular fluid loss by isotonic dehydration associated with manifest or imminent acidosis' in all the Nordic countries (Summary of Product Characteristics for Sterofundin, B Braun Medical).

### Inclusion

All adult patients

• who need **fluid resuscitation in the ICU**

• AND who have fulfilled the criteria for **severe sepsis **within the previous 24 hours according to the Society of Critical Care Medicine/American College of Chest Physicians (SCCM/ACCP), see Additional file 1[20]

• AND where informed **consent is obtainable **either from the patient or by proxy (In Denmark: Two physicians followed by delayed consent from next of kin and the patient's general practitioner. In Iceland, Finland and Norway: Next of kin)

The following patients will not be included:

• Age < 18 years

• Previously randomised in the 6S trial

• Allergy towards hydroxyethyl starch or malic acid

• Treatment with > 1000 ml of any synthetic colloid within the last 24 hours prior to randomisation

• Any form of renal replacement therapy

• Acute burn injury > 10% body surface area

• Severe hyperkalaemia, p-K > 6 mM

• Liver or kidney transplantation during current hospital admission

• Intracranial bleeding within current hospitalisation

• Enrolment into another ICU trial of drugs with potential action on circulation, renal function or coagulation

• Withdrawal of active therapy

### Randomisation

**Figure 1:**
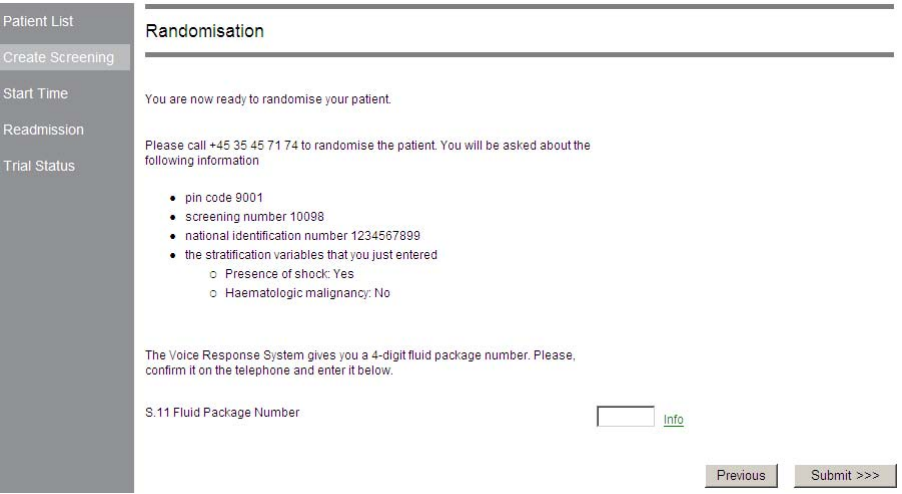
**Screen print of the randomisation instruction generated by the web-based electronic case record form following successful screening of a patient (all inclusion criteria fulfilled, no exclusion criteria fulfilled and consent obtained)**. Staff at trial sites will use this instruction to call the phone-based, interactive, voice response randomisation system available around the clock at Copenhagen Trial Unit for immediate and concealed allocation of the patient to trial fluid.

Staff at trial sites will have 24 hour access to a phone-based, interactive, voice response randomisation system at an independent trial unit (Copenhagen Trial Unit) to allow immediate and concealed allocation and intervention with trial fluid (see the randomisation instruction generated by the eCRF, Figure 1). Randomisation to trial fluid is performed in blocks (presence of shock or not, haematological malignancy or not and inclusion in a university hospital or not) with varying block sizes according to the generation of the allocation sequence by a computer at CTU. A unique identification number of the patient will have to be entered into the system to ensure that the same patient is not randomised twice. In addition, each patient will be given a unique patient-number and a randomisation number.

### Primary outcome measure

The composite outcome measure of 90-day mortality or end-stage kidney disease defined as dialysis-dependency 90 days after randomisation [21] will be the primary outcome measure, and these two outcome measures will also be analysed separately.

### Secondary outcome measures

• Twenty-eight-day, 6-month and 1-year mortality

• Sepsis-related organ failure assessment (SOFA, modified from [22], see Additional file 2) score (excluding Glasgow Coma Score) at day 5 after randomisation

• Kidney failure (SOFA score > 2 in the kidney component) at any time in the ICU after randomisation

• Development of kidney failure (doubling of p-creatinine values [3,4]) in the ICU after randomisation

• Development of acidosis as lowest recorded arterial blood pH in the ICU after randomisation

• Need of renal replacement therapy within 90 days after randomisation

• Need of mechanical ventilation within 90 days after randomisation

• Days alive without renal replacement therapy in 90 days after randomisation

• Days alive without mechanical ventilation in 90 days after randomisation

• Hospital length of stay for survivors sanctioned at 90 days after randomisation

### Interventions

**Figure 2:**
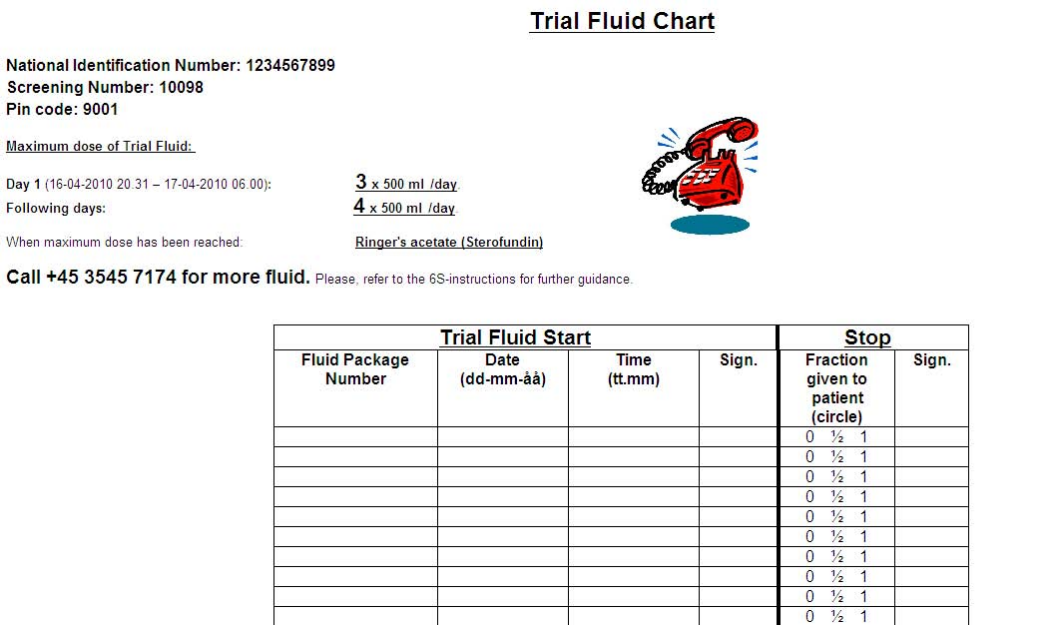
**Trial fluid chart generated by the web-based electronic case record form following successful randomisation of a patient**. The fluid chart is to be used bed-side by clinical staff to ensure proper use of trial fluid and document the administration of trial fluid.

Trial fluid is to be used for volume expansion during the entire ICU-stay for a maximum of 90 days. The treatment will follow the recommendations for fluid therapy given by the Surviving Sepsis Campaign, see Additional file 3[1]. The maximum dose of HES 130/0.4 recommended by the manufacturers is 50 ml/kg ideal BW/24 hours. The maximum dose of trial fluid will be 33 ml/kg ideal BW/24 hours as a recent retrospective study indicated that a dose of HES 130/0.4 above this might increase the risk of kidney failure in septic patients [23]. After that unmasked treatment with Ringer's acetate will be given to all patients (see the trial fluid chart generated by the eCRF, Figure 2). Maintenance fluids and nutrition should be given as clinically indicated. Blood products should be given for specific indications only as recommended by the Surviving Sepsis Campaign, see Additional file 3[1]. The treating clinicians will decide all other interventions. As this is a pragmatic trial, concomitant medication will not be registered except for use of potentially nephrotoxic drugs (see below).

The following will be done to reduce the risk of giving too high doses of trial fluid:

• The maximum daily dose of trial fluid will be based on estimated ideal body weight (men: estimated height in cm - 100; women: estimated height in cm - 105).

• The calculated maximum daily dose of trial fluid will be reduced to the nearest 500 ml.

• On the 1^st ^day of the trial, any synthetic colloids given 24 hours prior to randomisation will be subtracted the calculated dose of trial fluid.

If the patient is discharged and then readmitted to the ICU within 90 days of randomisation the allocated trial fluid must be used if volume expansion is indicated.

### Blinding

The trial fluids are visually identical and will be delivered in identical 500 ml 'flexibag' plastic bottles, which will be put in black plastic bags and sealed by trial personnel not involved in randomisation or treatment of patients. A computer program (CTU) will generate the coding list with the numbers for the bottles. At randomisation, the computer program (CTU) will allocate numbered bottles from the specific trial site to the patient. Each trial site will have sufficient number of bottles of trial fluid to be allocated to patients included. This will ensure that the patient only receives the trial fluid that he/she was randomised to receive.

### Safety

Patients will be withdrawn from the trial fluid-treatment protocol if

• Renal replacement therapy is commenced for acute kidney failure OR

• SARs or SUSARs occur (see below)

Patients withdrawn from the trial fluid-treatment protocol for the above reasons will receive open-label saline or Ringer's lactate for volume expansion for the remaining days of the 90-day study period.

The treating clinician can withdraw a patient from the trial fluid-treatment protocol if

• The clinical status of the patient requires open-label fluid treatment.

The independent Data Monitoring and Safety Committee - DMSC - will recommend pausing or stopping the trial if (see Additional file 4 for details)

• Group-difference in the primary outcome measure is found at the interim analysis

• Group-difference in 28- or 90-day mortality is found at the interim analysis

• Group-difference in SARs or SUSARs is found at the interim analysis

• Results from other trials show clear benefit or harm with one of the trial fluids

### Serious adverse reactions

The serious adverse reactions - SARs - described with the use of the trial fluids are allergic reactions (both) and bleeding (starch) (Tetraspan and Sterofundin Summaries of Product Characteristics). The occurrence of these will be recorded daily in the eCRF during the ICU stay and compared for the two trial groups by the DMSC at the interim analysis. An independent statistician will prepare the data for this. During the trial, Sponsor will send a monthly report on the occurrence of SARs to the DMSC and a yearly report to the ethics committees.

Suspected unexpected serious adverse reactions (SUSARs) will be defined as serious adverse reactions not described in the Summaries of Product Characteristics for Tetraspan and Sterofundin. SUSARs will be reported by trial site investigators to Sponsor through the eCRF within 24 h. Sponsor will report any SUSARs within 7 days to the medicines agency via EudraVigilance, to the DMSC, which may request the randomisation status of the patient, and to B. Braun Medical. During the trial, Sponsor will send a monthly report on the occurrence of SUSARs to the DMSC and a yearly report to the ethics committees.

Serious adverse events (SAEs) will not be recorded as an entity, because the majority of septic ICU patients will experience SAEs during their critical illness. The SAEs will be captured in the secondary outcome measures.

### Patient withdrawal

Patients who are withdrawn from the trial fluid-treatment protocol (see Safety) will be followed up and analysed as the remaining patients.

Patients may be withdrawn from the trial at any time if consent is withdrawn by the person(s), who has given surrogate consent or by the patient. The person making the withdrawal will be asked for permission to obtain data for the primary outcome measure. If this person declines, no more data will be collected (except in Denmark and Finland where data for the primary outcome measure will be collected centrally). All randomised patients will be reported, and all data available with consent will be used in the analyses. If appropriate, multiple imputation will be used [24]. If there are patients with missing data for the primary outcome measure, new patients will be randomised to obtain the full sample size.

Patients who are transferred to another ICU will be withdrawn from the trial fluid-treatment protocol unless this new ICU is an active trial site. If so the allocated trial fluid will be used if volume expansion is indicated in the new ICU. In any case, patients that are transferred to another ICU will be followed up for the primary outcome measure.

### Statistics

Analysis will be by intention-to-treat comparing the composite outcome measure of death or dialysis-dependency at 90 days in the two groups by chi-squared test (the primary analysis) and multiple logistic regression analysis adjusting for design and patient variables (stratification variables, age, diabetes, use of nephrotoxic drugs, previous renal dysfunction ('normal' p-creatinine > 100 μmol/l) [9], acute kidney failure at randomisation (kidney failure (SOFA score ≥ 2 in the kidney component) defining severe sepsis) [8] and SAPS II [9] and SOFA score [8] in the 24 h where randomisation was done according to the ICH guidelines [25].

2 × 400 patients will be needed to show a 20% RRR in the composite outcome measure under the assumption of a mortality of 45% (estimated from mean mortality rates in the AT III meta-analysis [26], the two groups in VISEP [4] and unpublished data from East Danish Septic Shock Cohort, A Perner, personal communication) and dialysis-dependency of 5% at 90 days [12,13], thus 50% for the composite outcome measure, with an alpha of 0.05 (two-sided) and a power of 80%. With this sample size an absolute reduction in the frequency of the composite outcome measure from 50% to 40% can be shown.

An interim analysis will be performed after 400 patients. The DMSC will recommend that the trial should be stopped if it finds a group difference in primary outcome measure, mortality, SARs or SUSARs with p ≤ 0.001 (Haybittle-Peto criteria) [27,28] or otherwise find that the continued conduct of the trial clearly compromises patient safety.

### Data registration

**Figure 3:**
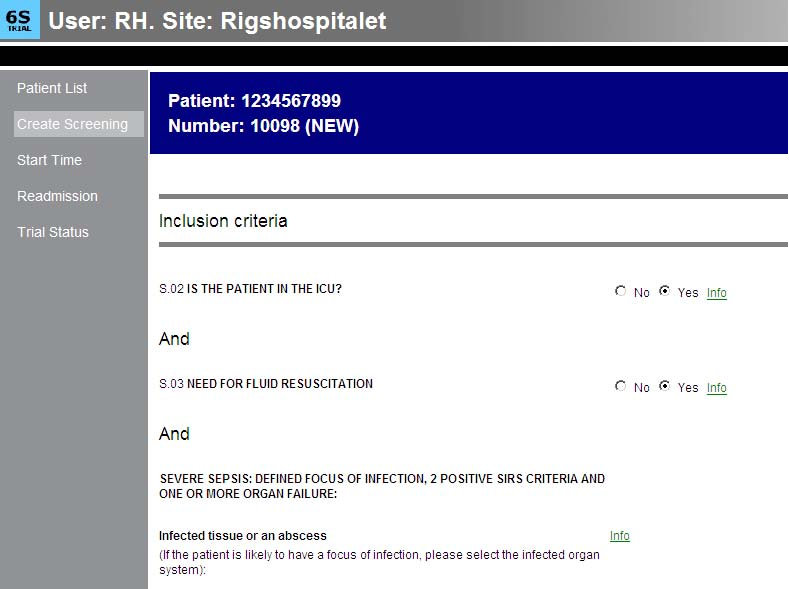
**Screen print of the first page of the screening part of the web-based electronic case record form (eCRF) (ExpertMaker, Lund, Sweden)**.

Data will be entered into the web-based electronic case record form (eCRF) (ExpertMaker, Lund, Sweden, Figure 3) from patient notes (source) by trial or clinical personnel under the supervision of the trial site investigators. From the eCRFs the trial database (CTU) will be established. Paper CRF will be used in case of technical difficulties with the eCRF.

The following data will be registered:

Pre-randomisation characteristics (all obtained from hospital notes):

National identification number, sex, age at randomisation, estimated height in cm, co-morbidities (previously admitted for heart failure, myocardial infarction, stroke or asthma or chronic obstructive pulmonary disease Y/N, chronic treatment for arterial hypertension or diabetes Y/N, haematological malignancy Y/N, metastatic cancer Y/N, AIDS Y/N, from where was the patient admitted to the ICU? (emergency ward/general ward/operation theatre or recovery/via paramedic or ambulance services/other ICU this hospital/other hospital), elective or emergency surgery within current hospital admission Y/N, site of infection (pulmonary/abdominal/urinary tract/soft tissue/other), and habitual p-creatinine. Use of potential nephrotoxic drugs (Y/N) during current hospital admission: IV gentamycin, IV vancomycin, IV amphotericin B, IV polymyxins, ciclosporin A, IV contrast dye, NSAIDs and Cox-2 inhibitors.

24-hours prior to randomisation:

• Values for simplified acute physiology score (SAPS) II [29]

• Volume of resuscitation fluids (crystalloids, colloids and blood products specified in ml)

• Results of blood samples (standard lab. values) for haemoglobin (lowest value), bilirubin (highest value), INR (highest value), d-dimer (highest value) and platelets (lowest value)

• Variables for SOFA scoring not covered above: Lowest mean arterial blood pressure value and highest infusion rate of vasoactive drugs

At randomisation (+/- 2 hours):

• Highest heart rate, lowest values of mean arterial blood pressure, central venous pressure, central venous oxygen saturation and highest values of arterial or venous lactate concentration obtained from ICU charts

12-hourly in the first 24 after randomisation:

• Highest and lowest values of central venous pressure, central venous oxygen saturation and arterial or venous lactate concentration obtained from ICU charts

Daily in the first 5 days after randomisation:

• Results of morning samples of bilirubin, INR and platelets (standard lab. values).

• Variables for daily SOFA scoring not covered above: Lowest blood pressure value and highest infusion rate of vasoactive drugs and lowest ratio of arterial oxygen tension/fraction of inspired oxygen obtained from the ICU charts

During the entire ICU stay:

• Daily volumes of trial fluid (reduced to aliquots of 250 ml) and other fluids including blood products, nutrition, total fluid input, urinary output and calculated fluid balance as of the ICU charts

• Daily lowest values of blood haemoglobin and arterial pH and standard base excess (point of care testing) and highest value of p-creatinine (standard lab. value)

• On mechanical invasive- or non-invasive ventilation (as marked in the SOFA scoring day 0-5 and Y/N at 08.00 from day 6)

• Renal replacement therapy on that day Y/N

• Any use of potential nephrotoxic drugs as mentioned above (Y/N for every day)

• Bleeding episodes noted in patient files including gastrointestinal (haematemesis, frank blood or "coffee grounds" in a nasogastric aspirate, or melaena or frank blood in stools), wounds, during surgery or frank blood in urine or tracheal aspirates.

• Serious adverse reactions (Y/N for every day) including severe bleeding (intracranial bleeding or bleeding episode (defined as above) with the need for ≥ 3 units of blood per day (defined as the 24 h of the units fluid charge)) or serious allergic reactions defined as urticaria associated with worsened circulation (20% decrease in blood pressure or 20% increase in vasopressor dose), increased airway resistance (20% increase in the peak pressure on the ventilation or clinical stridor or bronchospasme or treatment with bronchodilators).

90 days after randomisation:

• Survival status obtained from hospital or civil registries

• If the patient is deceased, date of death

• Dialysis-dependency at day 90 defined as need of renal replacement therapy within the time period 4 days prior to or after day 90 post-randomisation as obtained from hospital notes or registries.

• Total days of dialysis-dependency (any form of haemo-dialysis or -filtration) summarised at day 90 from hospital notes or registries. First and last treatment session will define length of dialysis-dependency in each patient.

• Date of hospital discharge as obtained from hospital notes or registries

1/2- and 1 year after randomisation:

• Survival status obtained from hospital or civil registries

### Data handling and retention

Data will be handled according to the data protection agencies of the different countries. All original records (including consent forms, CRFs, SUSAR reports and relevant correspondences) will be retained at trial sites or CTU for 15 years to allow inspection by the GCP Unit or local authorities. The study database will be maintained for 15 years and anonymised if requested by the authorities.

### Monitoring

The trial will be externally monitored (the GCP unit at University of Copenhagen) to GCP standards according to the EU directive 2001/20. Trial site investigators will give access to source data according to the Clinical Trial Agreement.

### Ethics

The trial will adhere to the Helsinki Declaration II and the national laws in the Nordic countries. Inclusion will start after approval by the ethical committees, medicines agencies and data protection agencies in the country of the trial site and trial registration at http://www.clinicaltrials.gov.

Patients will only be enrolled after informed consent, but the treatment has to be initiated immediately and most patients will be unconscious and therefore included after proxy consent according to national laws (In Denmark: Two physicians followed by delayed consent from next of kin and the patient's general practitioner. In Iceland, Finland and Norway: Next of kin).

The trial cannot be performed in conscious persons, as no clinically relevant model of severe sepsis exists and no conscious patients have the combination of severe infection and multiple organ failure as septic patients have.

No biological material will be collected for the trial, thus no bio-bank will be formed.

### Enrolment

The trial was registered 2009-08-09. Patients are expected to be included from 30 Scandinavian ICUs (Denmark 18 units, Sweden 5, Norway 3, Finland 3 units and Iceland 1 unit) during a 2-year period starting 2009-12-23.

Each unit has to include 2 patients per month (holidays excluded) to finish inclusion in 2 years.

Presently (2010-08-15), 14 ICUs are active trial sites; 259 patients have been screened and 155 randomised.

### Data analyses and publications

An independent statistician will perform the data analysis prior to the breaking of the randomisation code. Based on these masked analyses of the primary and secondary outcome measures two abstracts with conclusions will be written by the Writing Committee, the randomisation code will then be opened and a final manuscript written containing the correct of the two pre-made abstracts. The manuscript will be submitted to one of the major clinical journals regardless of the results. The Steering committee will grant authorship depending on personal input (see Additional file 5) according to the Vancouver definitions. All trial sites and trial site investigators will be acknowledged. Funding sources will have no influence on data handling or analysis or writing of the manuscript. Side studies will be allowed if supported by the Steering committee.

### Timeline

2008 - 2009: Applications for funding, ethical committees and medicines agencies, development of CRF and data management tools and development of monitoring plan and education of monitors

2010 - 2011: Inclusion of patients

Mid 2011: Interim analysis

Early 2012: Data analyses, writing and submission of the main manuscript for publication

### Collaborators

Copenhagen Trial Unit, Centre for Clinical Intervention Research, Rigshospitalet has developed the CRF together with the Steering Committee and systems for phone-based randomisation, allocation of trial fluid and data handling.

The GCP unit at University of Copenhagen has developed the monitoring plan and coordinated the monitoring in collaboration with the GCP units in Aarhus, Odense, Lund and Oslo.

The Scandinavian Society of Anaesthesia and Intensive Care provides the platform for web-based data entry - the eCRF (ExpertMaker AB, Lund, Sweden).

B Braun Medical AG (Melsungen, Germany) delivers trial fluids to all trial sites.

### Finances

The trial is fully funded by the Danish Strategic Research Council, the Danish Research Council, Rigshospitalet, the ACTA foundation and resources at trial sites. The funding sources have no influence on the trial design or data collection, management, analysis or reporting. The patients are covered by local insurance at the trial sites.

### Perspective

Severe sepsis affects millions of patients worldwide with high rates of complications and mortality. Outcome differences between therapies for severe sepsis will, therefore, have major impact on global health and healthcare costs.

Currently, three other RCTs are assessing the effects of HES 130/0.4 in sepsis:

BASES is a single centre study expected to include 250 patients with severe sepsis. The primary outcome measure is ICU length of stay, but mortality and the frequency of renal replacement therapy is also recorded (clinicaltrial.gov identifier: NCT00273728). Recruitment is expected to be completed in May 2010 (M Siegemund, personal communication).

CRYSTMAS is a multicentre study expected to include 200 patients with severe sepsis. The primary outcome measure is amount of fluid required to achieve initial haemodynamic stabilisation and amount of enteral calories given in the 7 days after stabilisation, but markers of organ failure will also be recorded. Recruitment has been stopped in March 2010 (clinicaltrial.gov identifier: NCT00464204).

CHEST is an Australian/New Zealand multicentre RCT comparing HES 130/0.4 and saline in 7,000 hypovolaemic ICU patients (clinicaltrial.gov identifier: NCT00935168). The primary outcome measure is 90-day mortality and subgroup analysis of patients with severe sepsis is planned (J Myburgh, personal communication). We collaborate with the investigators and plan a common individual patient data meta-analysis combining our data with those of their patients with severe sepsis (1,000 expected). Thus inclusion and exclusion criteria and outcome measures of the two trials have been harmonised to prepare for the common analysis.

Even though none of the trials alone may be definitive in answering the questions regarding safety and efficacy of HES 130/0.4 in patients with severe sepsis, the results will form the basis for meta-analysis of data from more than 2,000 septic patients.

## Discussion

The 6S trial will provide important safety and efficacy data on the use of HES 130/0.4 in patients with severe sepsis.

## Competing interests

The authors declare that they have no competing interests.

## Authors' contributions

All authors made substantive contributions to the 6S trial as trial site investigators and revised and gave final approval of the manuscript. AP drafted the manuscript together with NH and JW. AP is the principal investigator and sponsor of 6S and designed the trial together with NH, JW, AÅ, ABG, JT, and GK. NH is the trial manager and member of the steering and management committee. AP, AÅ, ABG, JT and GK are national investigators. VP, ER, SiK and JaW are national co-investigators.

## Supplementary Material

Additional file 1 Trial criteria for severe sepsis Click here for file

Additional file 2 Severity organ failure assessment scoring in the 6S trial Click here for file

Additional file 3 The Surviving Sepsis Campaigns recommendations on fluid and blood product therapy Click here for file

Additional file 4 Charter for the independent Data Monitoring and Safety Committee (DMSC) of the 6S trial Click here for file

Additional file 5 Acknowledgment of academic contribution and authorship Click here for file
