# Supplementary material for: Comparing the effect of hydroxyethyl starch 130/0.4 with balanced crystalloid solution on mortality and kidney failure in patients with severe sepsis (6S - Scandinavian Starch for Severe Sepsis/Septic Shock trial): Study protocol, design and rationale for a double-blinded, randomised clinical trial
Source: Trials. 2011 Jan 27;12:24. doi: 10.1186/1745-6215-12-24 (PMC3040153; doi:10.1186/1745-6215-12-24)
Supplement: Additional file 1 — Trial criteria for severe sepsis [file 1745-6215-12-24-S1.DOC]

**Additional file 1**

Trial criteria for severe sepsis

**SEPSIS** is defined as a (1) DEFINED FOCUS OF INFECTION AND (2) at least TWO systemic inflammatory response syndrome (SIRS) criteria.

(1) DEFINED FOCUS OF INFECTION is indicated by either

(i) An organism grown in blood or sterile site

OR

(ii) An abscess or infected tissue (e.g. pneumonia, peritonitis, urinary tract, vascular line infection, soft tissue, etc).

(2) The 4 SIRS criteria are:

1. CORE TEMPERATURE >38°C or <36°C. (Core temperature is rectal, urinary bladder, central line, or tympanic). If oral, inguinal or axillary temperatures are used, add 0.5°C to the measured value. Hypothermia <36°C must be confirmed by core temperature only. Use the most deranged value recorded in the 24 hours before randomisation.
2. HEART RATE >90 beats/minute. If patient had an atrial arrhythmia, record the ventricular rate. If patients have a known medical condition or are receiving treatment that would prevent tachycardia (for example, heart block or beta blockers), they must meet two of the remaining three SIRS criteria. Use the most deranged value recorded in the 24 hours before randomisation.
3. RESPIRATORY RATE > 20 breaths per minute or a PaCO_2_ < 4.3 kPa (32 mmHg) or mechanical ventilation for an acute process. Use the most deranged respiratory rate or PaCO_2_ recorded in the 24 hours before randomisation.
4. WHITE BLOOD CELL COUNT of >12 x 10^9^/l or < 4 x 10^9^/l or > 10% immature neutrophils (band forms). Use the most deranged value recorded in the 24 hours before randomisation.

**SEVERE SEPSIS** is defined as SEPSIS plus at least ONE ORGAN FAILURE, except when that organ failure was already present 48 hours before the onset of sepsis.

ORGAN FAILURE is defined as a Sequential Organ Failure Assessment (SOFA) score > 2 for the organ in question, see Additional file 5.
