# Supplementary material for: Comparing the effect of hydroxyethyl starch 130/0.4 with balanced crystalloid solution on mortality and kidney failure in patients with severe sepsis (6S - Scandinavian Starch for Severe Sepsis/Septic Shock trial): Study protocol, design and rationale for a double-blinded, randomised clinical trial
Source: Trials. 2011 Jan 27;12:24. doi: 10.1186/1745-6215-12-24 (PMC3040153; doi:10.1186/1745-6215-12-24)
Supplement: Additional file 2 — Severity organ failure assessment scoring in the 6S trial [file 1745-6215-12-24-S2.DOC]

### Additional file 2: SOFA scoring (ex. GCS) - use the most deranged value recorded in the previous 24 h [22]

| **ORGAN SYSTEM** | **0** | **1** | **2** | **3** | **4** | Organ scores |
| --- | --- | --- | --- | --- | --- | --- |
| Respiration |  |  |  |  |  |  |
| PaO_2_ / FiO_2_ (in mmHg) | >400 | 301 - 400 | <301 (without respiratory support*) | 101 – 200 (with respiratory support*) | ≤ 100 (with respiratory support*) |
| (in kPa) | >53 | 40 – 53 | <40 (without respiratory support*) | 13 – 27 (with respiratory support*) | ≤ 13 (with respiratory support*) |
| Coagulation Platelets (x 10^9^/l) | >150 | 101 - 150 | 51 - 100 | 21 - 50 | ≤ 20 |  |
| Liver |  |  |  |  |  |  |
| Bilirubin (mg/dl) | < 1.2 | 1.2 – 1.9 | 2.0 – 5.9 | 6.0 – 11.9 | > 12.0 |
| (μmol/l) | <20 | 20 - 32 | 33 - 101 | 102 - 204 | >204 |
| Cardiovascular Hypotension | Mean arterial pressure  (MAP) > 70 mmHg | MAP < 70 mmHg | dopamine ≤ 5.0 (dose in μg/kg/min) | dopamine > 5.0  (dose in μg/kg/min) | dopamine > 15.0  (dose in μg/kg/min) |  |
|  |  |  | or any dose dobutamine | or adrenalin ≤ 0.1 | or adrenalin >0.1 |
|  |  |  | or any dose milrinone  or any dose levosimendan | or noradrenalin ≤ 0.1  or any dose vaso-pressin or any dose phenylephrine | or noradrenalin >0.1 |
| **Renal**  Creatinine (mg/dl) | < 1.2 | 1.2 – 1.9 | 2.0 – 3.4 | 3.5 – 4.9 | > 5.0 |  |
| (μmol/l) | < 110 | 110 – 170 | 171 – 299 | 300 – 440 | > 440 |
| **OR** Urine output |  |  |  | or < 500 ml / day | or < 200 ml / day |

If a value has not been measured, the score 0 should be given. *Respiratory support is defined as any form of invasive or non-invasive ventilation including continuous positive airway pressure delivered through mask or tracheotomy
