# Supplementary material for: Comparing the effect of hydroxyethyl starch 130/0.4 with balanced crystalloid solution on mortality and kidney failure in patients with severe sepsis (6S - Scandinavian Starch for Severe Sepsis/Septic Shock trial): Study protocol, design and rationale for a double-blinded, randomised clinical trial
Source: Trials. 2011 Jan 27;12:24. doi: 10.1186/1745-6215-12-24 (PMC3040153; doi:10.1186/1745-6215-12-24)
Supplement: Additional file 3 — The Surviving Sepsis Campaigns recommendations on fluid and blood product therapy [file 1745-6215-12-24-S3.DOC]

**Additional file 3**

The Surviving Sepsis Campaigns recommendations [1]

For fluid therapy

- Use a fluid challenge technique while associated with a haemodynamic improvement
- Give fluid challenges of 500 - 1000 ml over 30 min. More rapid and larger volumes may be required in sepsis-induced tissue hypoperfusion
- Rate of fluid administration should be reduced if cardiac filling pressures increase without concurrent haemodynamic improvement

For administration of blood products

- Give red blood cells when haemoglobin decreases to < 7.0 g/dl (< 4.5 mM) to target haemoglobin of 7.0 - 9.0 g/dl (4.5 – 5.6 mM). A higher haemoglobin level may be required in special circumstances (e.g.: myocardial ischaemia, severe hypoxaemia, acute haemorrhage, cyanotic heart disease or lactic acidosis)
- Do not use fresh frozen plasma to correct laboratory clotting abnormalities unless there is bleeding or planned invasive procedures
- Administer platelets when
  - counts are < 5 × 10^9^/l regardless of bleeding
  - counts are 5 - 30 × 10^9^/l and there is significant bleeding risk
  - higher platelet counts (≥ 50 × 10^9^/l) are required for surgery or invasive procedures
