# Supplementary material for: Comparing the effect of hydroxyethyl starch 130/0.4 with balanced crystalloid solution on mortality and kidney failure in patients with severe sepsis (6S - Scandinavian Starch for Severe Sepsis/Septic Shock trial): Study protocol, design and rationale for a double-blinded, randomised clinical trial
Source: Trials. 2011 Jan 27;12:24. doi: 10.1186/1745-6215-12-24 (PMC3040153; doi:10.1186/1745-6215-12-24)
Supplement: Additional file 4 — Charter for the independent Data Monitoring and Safety Committee (DMSC) of the 6S trial [file 1745-6215-12-24-S4.DOC]

**Additional file 4**

Charter for the independent Data Monitoring and Safety Committee (DMSC) of the 6S trial.

EudraCT no. 2009-010104-28 Clinical Trial no. NCT00962156.

Copenhagen 2009

**Introduction**

The Charter will define the primary responsibilities of the DMSC, its relationship with other trial components, its membership, and the purpose and timing of its meetings. The Charter will also provide the procedures for ensuring confidentiality and proper communication, the statistical monitoring guidelines to be implemented by the DMSC, and an outline of the content of the Open and Closed Reports that will be provided to the DMSC.

## Primary responsibilities of the DMSC

The DMSC will be responsible for safeguarding the interests of trial participants, assessing the safety and efficacy of the interventions during the trial, and for monitoring the overall conduct of the clinical trial. The DMSC will provide recommendations about stopping or continuing the trial to the Steering Committee (SC). To contribute to enhancing the integrity of the trial, the DMSC may also formulate recommendations relating to the selection/recruitment/retention of participants, their management, improving adherence to protocol-specified regimens and retention of participants, and the procedures for data management and quality control.

The DMSC will be advisory to the SC. The SC will be responsible for promptly reviewing the DMSC recommendations, to decide whether to continue or terminate the trial, and to determine whether amendments to the protocol or changes in trial conduct are required.

## Members of the DMSC

The DMSC is an independent multidisciplinary group consisting of clinicians and a biostatistician that, collectively, has experience in the management of ICU patients and in the conduct, monitoring and analysis of randomised clinical trials.

# DMSC Clinician

Dr. Daniel De Backer, Department of Intensive Care, Erasme University Hospital, Brussels

DMSC Clinical trialist

Prof. Kathy Rowan, Intensive Care National Audit and Research Center (ICNARC), London

# DMSC Biostatistician

Dr. Peter Dalgaard, Department of Biostatistics, University of Copenhagen

## Conflicts of interest

DMSC membership has been restricted to individuals free of conflicts of interest. The source of these conflicts may be financial, scientific, or regulatory in nature. Thus, neither trial investigators nor individuals employed by the sponsor, nor individuals who might have regulatory responsibilities for the trial products, are members of the DMSC. The DMSC members do not own stock in the companies having products being evaluated by the 6S trial.

The DMSC members will disclose to fellow members any consulting agreements or financial interests they have with the sponsor of the trial, with the contract research organisation (CRO) for the trial (if any), or with other sponsors having products that are being evaluated or having products that are competitive with those being evaluated in the trial.

The DMSC will be responsible for deciding whether these consulting agreements or financial interests materially impact their objectivity.

The DMSC members will be responsible for advising fellow members of any changes in these consulting agreements and financial interests that occur during the course of the trial. Any DMSC members who develop significant conflicts of interest during the course of the trial should resign from the DMSC.

DMSC membership is to be for the duration of the clinical trial. If any members leave the DMSC during the course of the trial, the SC will appoint the replacement(s).

## Formal interim analysis meeting

One 'Formal Interim Analysis' meeting will be held to review data relating to treatment efficacy, patient safety, and quality of trial conduct. The three members of the DMSC will meet when 90-day follow-up data of 400 patients have been obtained.

## Proper communication

To enhance the integrity and credibility of the trial, procedures will be implemented to ensure the DMSC has sole access to evolving information from the clinical trial regarding comparative results of efficacy and safety data, aggregated by treatment group (0,1). An exception will be made to permit access to an independent statistician who will be responsible for serving as a liaison between the database and the DMSC.

At the same time, procedures will be implemented to ensure that proper communication is achieved between the DMSC and the trial investigators. To provide a forum for exchange of information among various parties who share responsibility for the successful conduct of the trial, a format for Open Sessions and Closed Sessions will be implemented. The intent of this format is to enable the DMSC to preserve confidentiality of the comparative efficacy results while at the same time providing opportunities for interaction between the DMSC and others who have valuable insights into trial-related issues.

Closed Sessions

Sessions involving only DMSC membership who generates the Closed Reports (called Closed Sessions) will be held to allow discussion of confidential data from the clinical trial, including information about the relative efficacy and safety of interventions. To ensure that the DMSC will be fully informed in its primary mission of safeguarding the interest of participating patients, the DMSC will be blinded in its assessment of safety and efficacy data. However, the DMSC can request unblinding from the SC.

Open Reports

For each DMSC meeting, Open Reports will be provided available to all who attend the DMSC meeting. The Reports will include data on recruitment and baseline characteristics, and pooled data on eligibility violations, completeness of follow-up, and compliance. The primary trial statistician will prepare these Open Reports.

Closed Reports will include analysis of the primary efficacy outcome measure. In addition, analyses of the secondary outcome measures and serious adverse events will also be reported. These Closed Reports will be prepared by an independent biostatistician, with assistance from the trial biostatisticians, in a manner that allow them to remain blinded.

The Closed Reports should provide information that is accurate, with follow-up on mortality that is complete to within two months of the date of the DMSC meeting.

The Reports should be provided to DMSC members approximately three days prior to the date of the meeting.

Minutes of the DMSC Meetings

The DMSC will prepare minutes of their meetings. The Closed Minutes will describe the proceedings from all sessions of the DMSC meeting, including the listing of recommendations by the Committee. Because it is likely that these minutes may contain unblinded information, it is important that they are not made available to anyone outside the DMSC.

**Recommendations to the Steering Committee**

After the interim analysis meeting, the DMSC will make a recommendation to the SC to continue, hold or terminate the trial.

This recommendation will be based primarily on safety and efficacy considerations and will be guided by statistical monitoring guidelines defined in this Charter and the trial protocol.

The SC is jointly responsible with the DMSC for safeguarding the interests of participating patients and for the conduct of the trial. Recommendations to amend the protocol or conduct of the trial made by the DMSC will be considered and accepted or rejected by the SC. The SC will be responsible for deciding whether to continue, hold or stop the trial based on the DMSC recommendations.

The DMSC will be notified of all changes to the trial protocol or conduct. The DMSC concurrence will be sought on all substantive recommendations or changes to the protocol or trial conduct prior to their implementation.

## Statistical monitoring guidelines

The outcome variables are defined in the 6S trial protocol.

For the two intervention groups, the DMSC will evaluate data on:

The primary outcome measure

The composite outcome measure of death or dialysis-dependency 90 days after randomisation analysed together and separately

## The secondary outcome measures

28-day mortality

Severity organ failure assessment (SOFA) score at day 5 after randomisation

Kidney failure (SOFA score >2 in the kidney component) during the ICU stay

Need of dialysis

Days free of dialysis for survivors

Serious adverse reactions - SARs - (severe allergic reactions or severe bleeding) and suspected unexpected serious adverse reactions - SUSARs

The DMSC will be provided with these data from the Coordinating Centre as:

1. Number of patients randomised
2. Number of patients randomised per intervention group (0,1)
3. Number of patients stratified pr. stratification variable per intervention group (0,1)
4. Number of events, according to the outcomes, in the two groups

Based on evaluations of these outcomes, the DMSC will decide if they want further data from the Coordinating Centre and when next to perform analyses of the data.

For analyses, the data will be provided in one file as described below.

Based on the analyses of the primary outcome measure and SARs, the DMSC will use P<0.001 (Haybittle-Peto) [26,27] as the statistical limit to guide its recommendations regarding early termination of the trial.

Based on 28- and 90-day mortality analyses, the DMSC will use P<0.001 (Haybittle-Peto) and group sequential monitoring boundaries as the statistical limit to guide its recommendations regarding early termination of the trial.

DMSC should also be informed about all SUSARs and SARs occurring in the two groups of the trial.

The DMSC may also be asked to ensure that procedures are properly implemented to adjust trial sample size or duration of follow-up to restore power, if protocol specified event rates are inaccurate. If so, the algorithm for doing this should be clearly specified.

**Conditions for transfer of data from the Coordinating Centre to the DMSC**

The DMSC shall be provided with the data described below in one file.

The DMSC will be provided with an Excel database containing the data defined as follows:

1. Row 1 contains the names of the variables (to be defined below).
2. Row 2 to N (where N-1 is the number of patients who have entered the trial) each contains the data of one patient.
3. Column 1 to p (where p is the number of variables to be defined below) each contains in row 1 the name of a variable and in the next N rows the values of this variable.

The values of the following variables should be included in the database:

1: PtID: a number that uniquely identifies the patient.

2: Rdcode: The randomisation code (group 0 or 1) – the DMSC is not to be informed on what intervention the groups received.

3. 1.EndInd: Primary outcome measure indicator (1 if patient fulfilled the primary outcome measure at day 90 and 0 if the patient did not).

4: 90MInd: 90 day-mortality indicator (2 if patient is censored, 1 if patient was dead, and 0 if the patient was alive at day 90).

5: 90DIAInd: Dialysis dependency at day 90 (2 if patient is censored, 1 if patient is dependent, and 0 if the patient is not).

6: 28MInd: 28 day-mortality indicator (2 if patient is censored, 1 if patient is dead, and 0 if the patient is known to be alive).

7: SOFAInd: SOFA indicator (SOFA score of the patient at day 5).

8: AKFInd: Acute kidney failure indicator (1 if patient had kidney failure during the ICU stay, 0 if the patient did not).

9: DIAInd: Dialysis indicator (1 if patient needed any dialysis from randomisation till day 90 and 0 if the patient did not).

10: DiaDInd: Days free of dialysis indicator (no. of day from randomisation till day 90 free of dialysis for each patient; 0 if the patient did not need dialysis; blank if the patient had died).

11: SARInd: Serious Adverse Reaction indicator (1 if patient has had a SAR during ICU stay and 0 if the patient did not).

12: SUSARInd: Suspected Unexpected Serious Adverse Reaction indicator (1 if patient has had a SUSAR during ICU stay and 0 if the patient did not).
