# Supplementary material for: Comparing the effect of hydroxyethyl starch 130/0.4 with balanced crystalloid solution on mortality and kidney failure in patients with severe sepsis (6S - Scandinavian Starch for Severe Sepsis/Septic Shock trial): Study protocol, design and rationale for a double-blinded, randomised clinical trial
Source: Trials. 2011 Jan 27;12:24. doi: 10.1186/1745-6215-12-24 (PMC3040153; doi:10.1186/1745-6215-12-24)
Supplement: Additional file 5 — Acknowledgment of academic contribution and authorship [file 1745-6215-12-24-S5.DOC]

Additional file 5

## Acknowledgment of academic contribution and authorship

All trial sites including patients will be acknowledged, and all investigators at these sites will appear with their names under ‘the 6S trial investigators’ in an Additional file to the final manuscript.

The Steering Committee will grant authorship depending on personal involvement according to the Vancouver definitions. If a trial site investigator is to gain authorship, the site has to include 25 patients or more. If the site includes 50 patients or more, two authorships will be granted.

The listing of authors will be as follows: A Perner will be the first author, N Haase the second and the next authors will be the other members of the Steering Committee according to the number of included patients per country, then trial site investigators dependent on the number of included patients per site, J Wetterslev will appear as the last author and then ‘for the 6S trial investigators’.
